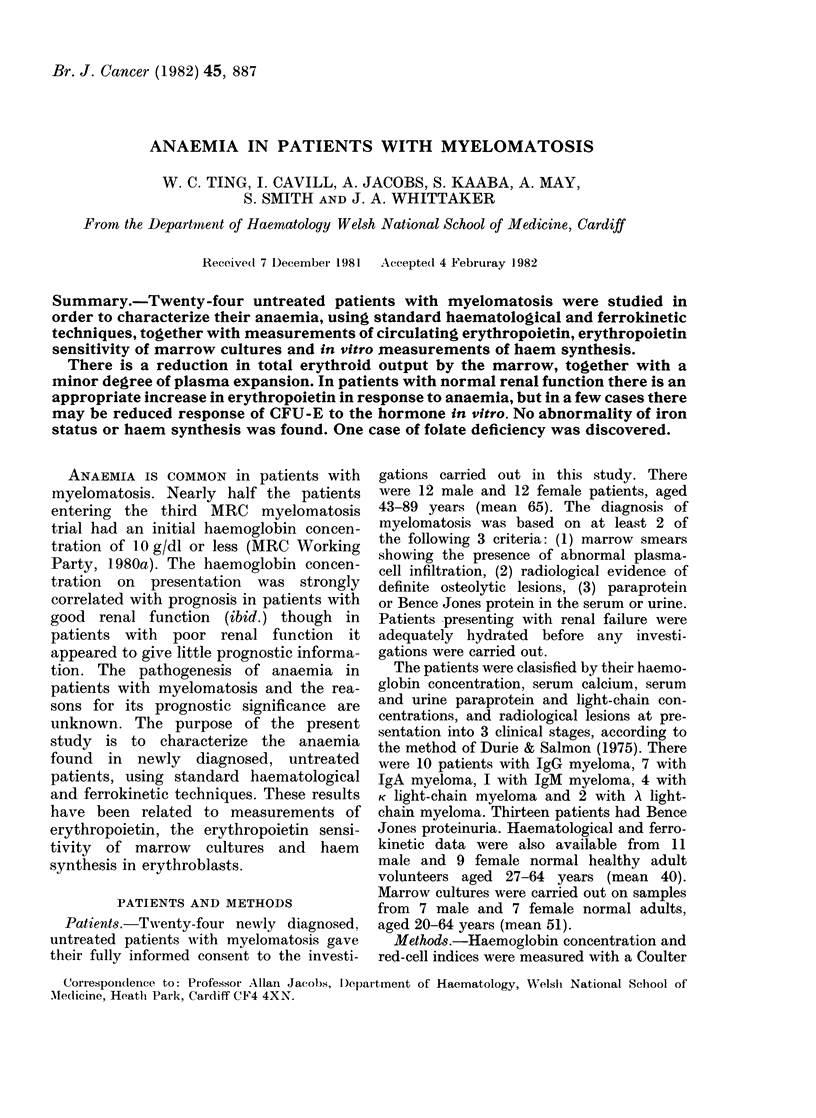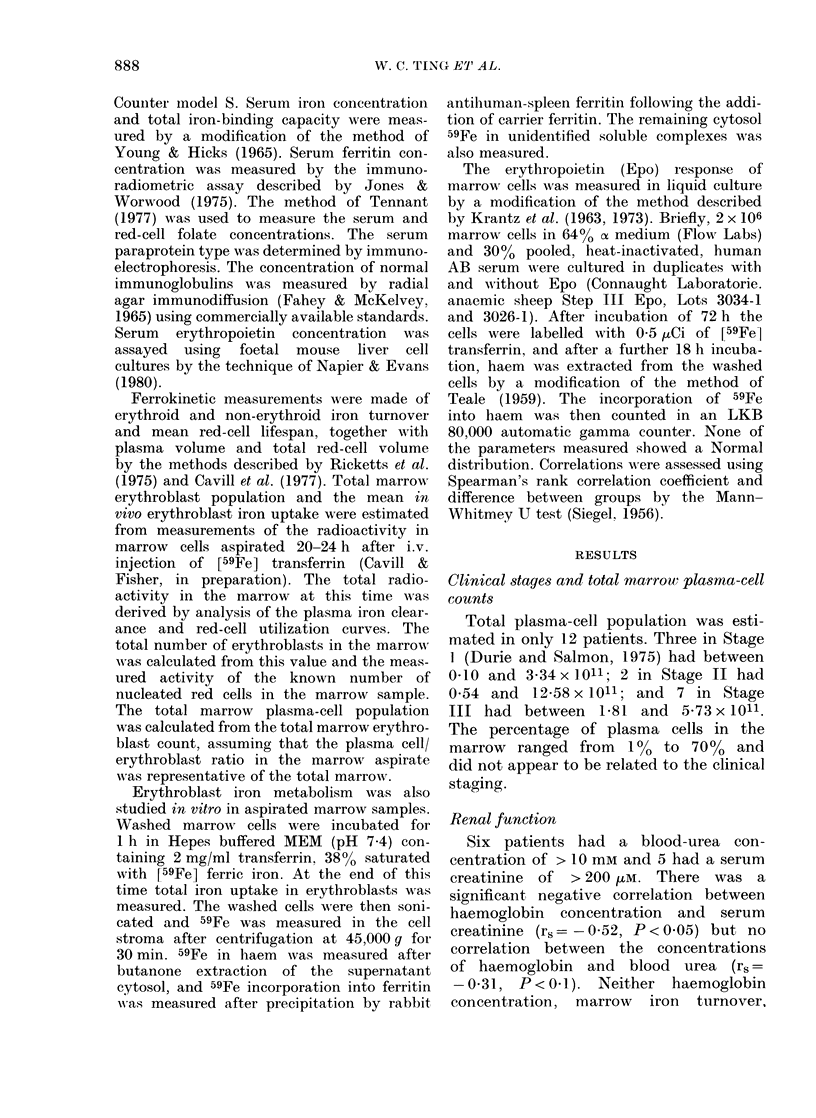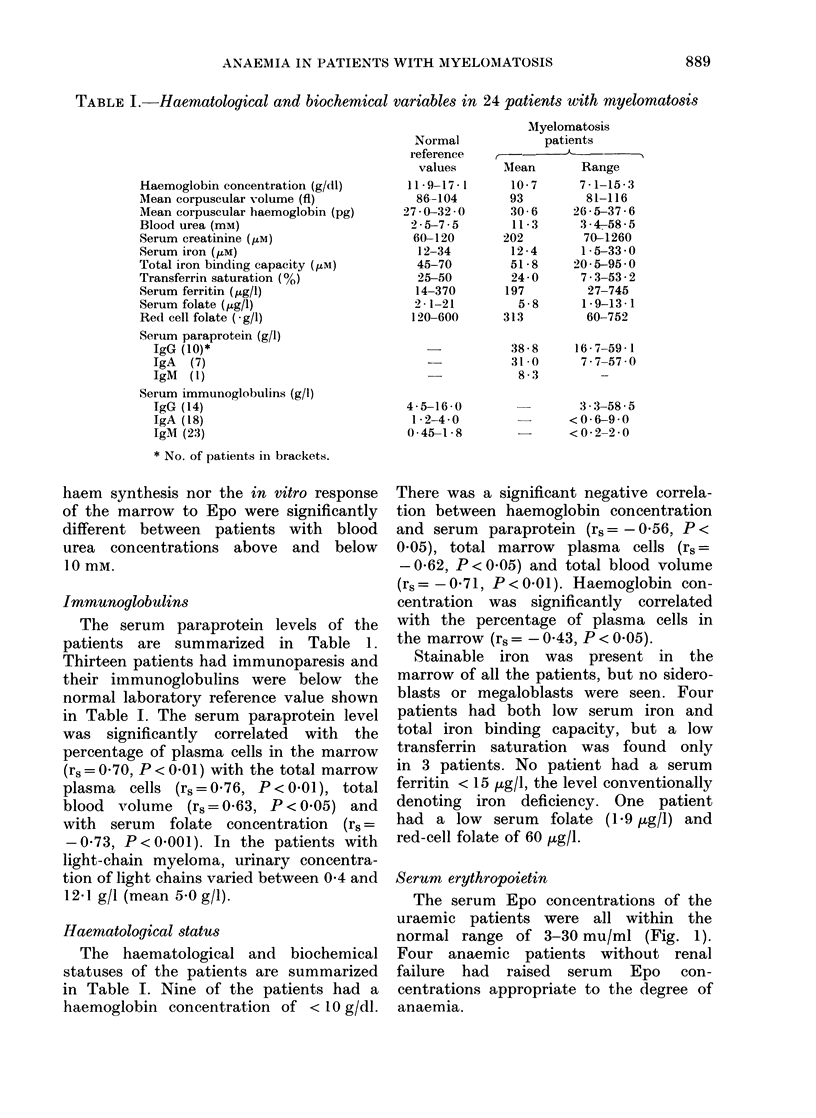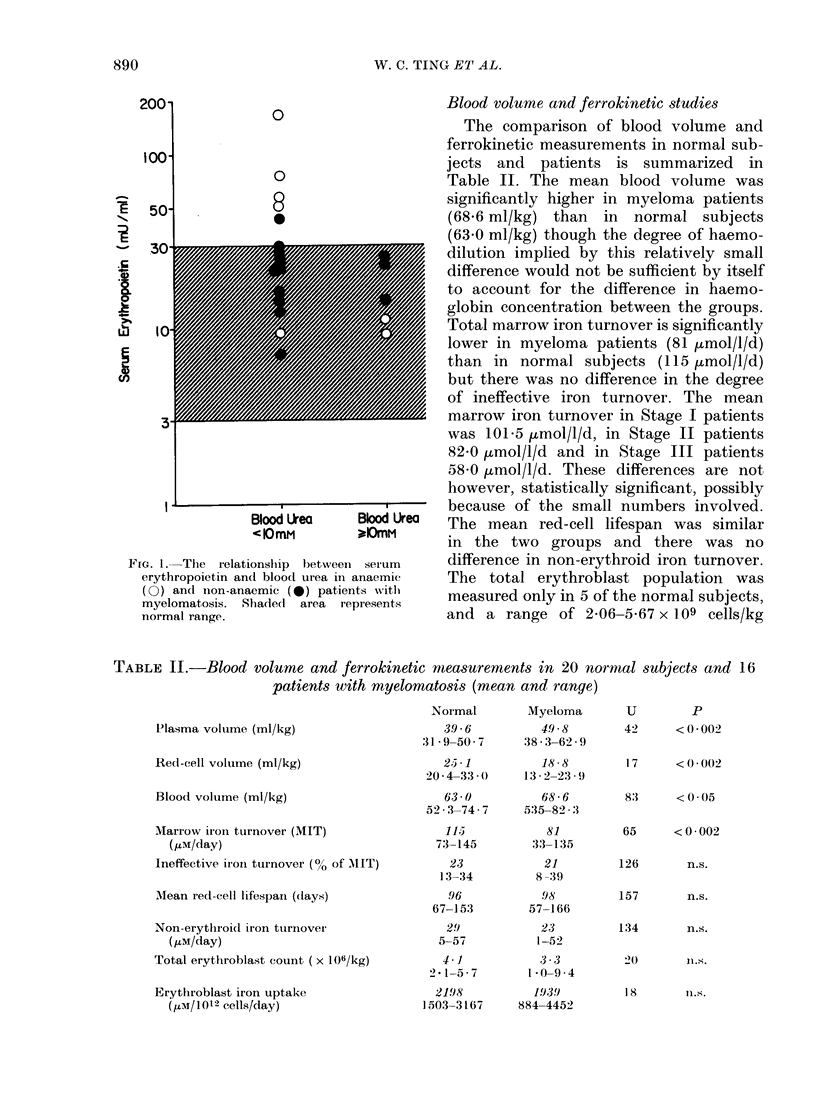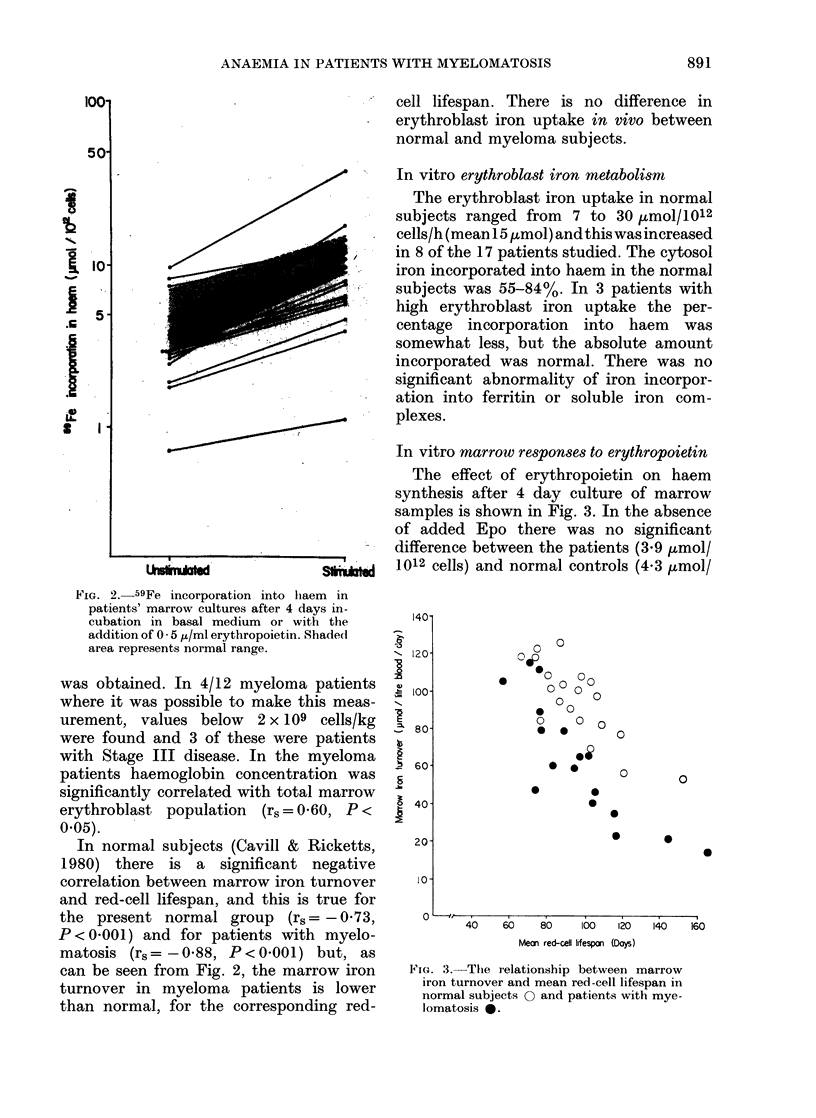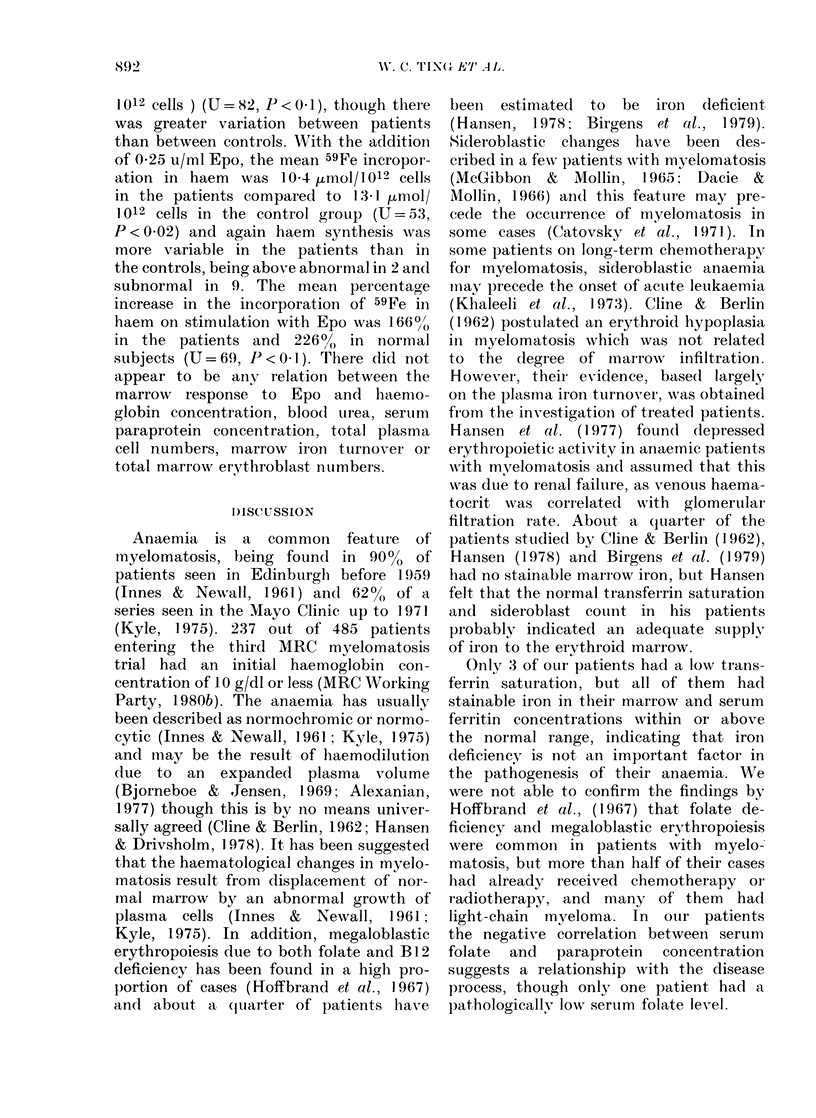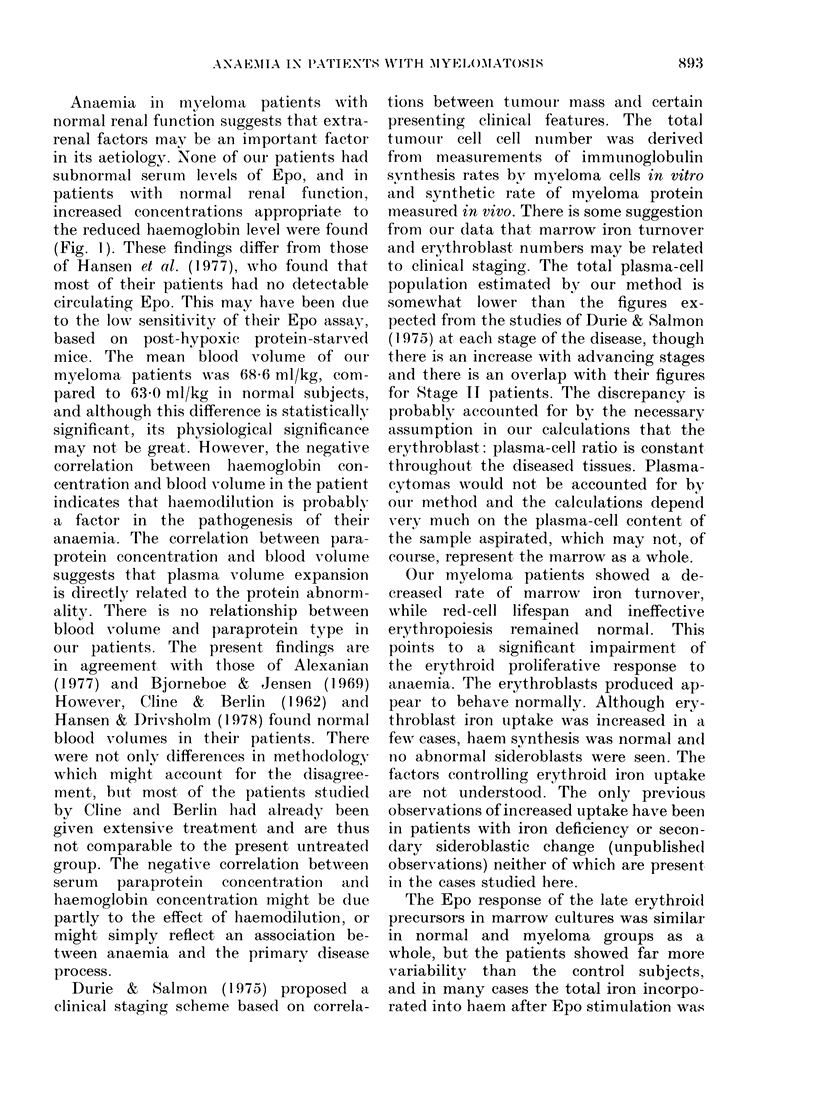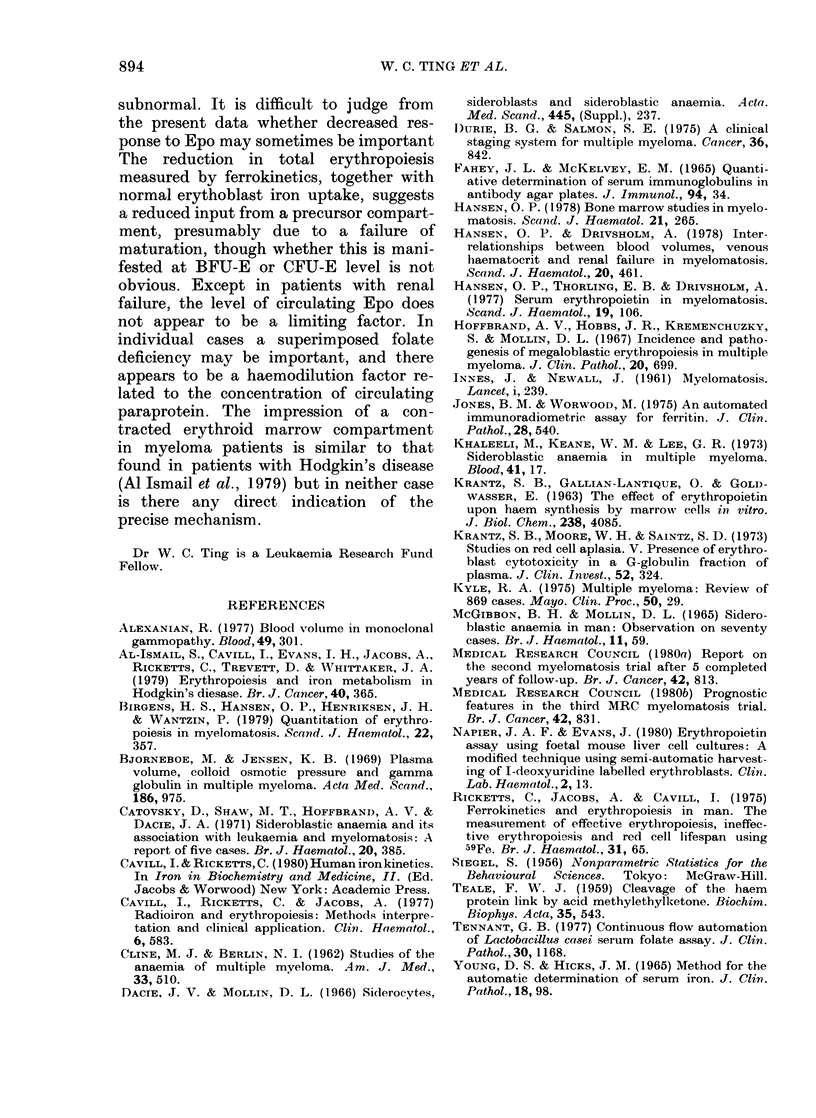# Anaemia in patients with myelomatosis.

**DOI:** 10.1038/bjc.1982.141

**Published:** 1982-06

**Authors:** W. C. Ting, I. Cavill, A. Jacobs, S. Kaaba, A. May, S. Smith, J. A. Whittaker

## Abstract

Twenty-four untreated patients with myelomatosis were studied in order to characterize their anaemia, using standard haematological and ferrokinetic techniques, together with measurements of circulating erythropoietin, erythropoietin sensitivity of marrow cultures and in vitro measurements of haem synthesis. There is a reduction in total erythroid output by the marrow, together with a minor degree of plasma expansion. In patients with normal renal function there is an appropriate increase in erythropoietin in response to anaemia, but in a few cases there may be reduced response of CFU-E to the hormone in vitro. No abnormality of iron status or haem synthesis was found. One case of folate deficiency was discovered.


*Br. J. Cancer* (1982) **45**, 887

### ANAEMIA IN PATIENTS WITH MYELOMATOSIS

W. C. TING, I. CAVILL, A. JACOBS, S. KAABA, A. MAY, S. SMITH AND J. A. WHITTAKER

*From the Department of Haematology Welsh National School of Medicine, Cardiff*

Received 7 December 1981 Accepted 4 Februray 1982

**Summary.—Twenty-four untreated patients with myelomatosis were studied in order to characterize their anaemia, using standard haematological and ferrokinetic techniques, together with measurements of circulating erythropoietin, erythropoietin sensitivity of marrow cultures and *in vitro* measurements of haem synthesis.**

**There is a reduction in total erythroid output by the marrow, together with a minor degree of plasma expansion. In patients with normal renal function there is an appropriate increase in erythropoietin in response to anaemia, but in a few cases there may be reduced response of CFU-E to the hormone *in vitro*. No abnormality of iron status or haem synthesis was found. One case of folate deficiency was discovered.**

ANAEMIA IS COMMON in patients with myelomatosis. Nearly half the patients entering the third MRC myelomatosis trial had an initial haemoglobin concentration of 10 g/dl or less (MRC Working Party, 1980*a*). The haemoglobin concentration on presentation was strongly correlated with prognosis in patients with good renal function (*ibid.*) though in patients with poor renal function it appeared to give little prognostic information. The pathogenesis of anaemia in patients with myelomatosis and the reasons for its prognostic significance are unknown. The purpose of the present study is to characterize the anaemia found in newly diagnosed, untreated patients, using standard haematological and ferrokinetic techniques. These results have been related to measurements of erythropoietin, the erythropoietin sensitivity of marrow cultures and haem synthesis in erythroblasts.

### PATIENTS AND METHODS

*Patients.*—Twenty-four newly diagnosed, untreated patients with myelomatosis gave their fully informed consent to the investigations carried out in this study. There were 12 male and 12 female patients, aged 43–89 years (mean 65). The diagnosis of myelomatosis was based on at least 2 of the following 3 criteria: (1) marrow smears showing the presence of abnormal plasma-cell infiltration, (2) radiological evidence of definite osteolytic lesions, (3) paraprotein or Bence Jones protein in the serum or urine. Patients presenting with renal failure were adequately hydrated before any investigations were carried out.

The patients were clasisfied by their haemoglobin concentration, serum calcium, serum and urine paraprotein and light-chain concentrations, and radiological lesions at presentation into 3 clinical stages, according to the method of Durie & Salmon (1975). There were 10 patients with IgG myeloma, 7 with IgA myeloma, I with IgM myeloma, 4 with $\kappa$ light-chain myeloma and 2 with $\lambda$ light-chain myeloma. Thirteen patients had Bence Jones proteinuria. Haematological and ferrokinetic data were also available from 11 male and 9 female normal healthy adult volunteers aged 27–64 years (mean 40). Marrow cultures were carried out on samples from 7 male and 7 female normal adults, aged 20–64 years (mean 51).

*Methods.*—Haemoglobin concentration and red-cell indices were measured with a Coulter

Correspondence to: Professor Allan Jacobs, Department of Haematology, Welsh National School of Medicine, Heath Park, Cardiff CF4 4XN.

Counter model S. Serum iron concentration and total iron-binding capacity were measured by a modification of the method of Young & Hicks (1965). Serum ferritin concentration was measured by the immunoradiometric assay described by Jones & Worwood (1975). The method of Tennant (1977) was used to measure the serum and red-cell folate concentrations. The serum paraprotein type was determined by immunoelectrophoresis. The concentration of normal immunoglobulins was measured by radial agar immunodiffusion (Fahey & McKelvey, 1965) using commercially available standards. Serum erythropoietin concentration was assayed using foetal mouse liver cell cultures by the technique of Napier & Evans (1980).

Ferrokinetic measurements were made of erythroid and non-erythroid iron turnover and mean red-cell lifespan, together with plasma volume and total red-cell volume by the methods described by Ricketts *et al.* (1975) and Cavill *et al.* (1977). Total marrow erythroblast population and the mean *in vivo* erythroblast iron uptake were estimated from measurements of the radioactivity in marrow cells aspirated 20–24 h after i.v. injection of [$^{59}$Fe] transferrin (Cavill & Fisher, in preparation). The total radioactivity in the marrow at this time was derived by analysis of the plasma iron clearance and red-cell utilization curves. The total number of erythroblasts in the marrow was calculated from this value and the measured activity of the known number of nucleated red cells in the marrow sample. The total marrow plasma-cell population was calculated from the total marrow erythroblast count, assuming that the plasma cell/erythroblast ratio in the marrow aspirate was representative of the total marrow.

Erythroblast iron metabolism was also studied *in vitro* in aspirated marrow samples. Washed marrow cells were incubated for 1 h in Hepes buffered MEM (pH 7·4) containing 2 mg/ml transferrin, 38% saturated with [$^{59}$Fe] ferric iron. At the end of this time total iron uptake in erythroblasts was measured. The washed cells were then sonicated and $^{59}$Fe was measured in the cell stroma after centrifugation at 45,000 *g* for 30 min. $^{59}$Fe in haem was measured after butanone extraction of the supernatant cytosol, and $^{59}$Fe incorporation into ferritin was measured after precipitation by rabbit antihuman-spleen ferritin following the addition of carrier ferritin. The remaining cytosol $^{59}$Fe in unidentified soluble complexes was also measured.

The erythropoietin (Epo) response of marrow cells was measured in liquid culture by a modification of the method described by Krantz *et al.* (1963, 1973). Briefly, $2 \times 10^6$ marrow cells in 64% $\alpha$ medium (Flow Labs) and 30% pooled, heat-inactivated, human AB serum were cultured in duplicates with and without Epo (Connaught Laboratorie. anaemic sheep Step III Epo, Lots 3034-1 and 3026-1). After incubation of 72 h the cells were labelled with 0·5 $\mu$Ci of [$^{59}$Fe] transferrin, and after a further 18 h incubation, haem was extracted from the washed cells by a modification of the method of Teale (1959). The incorporation of $^{59}$Fe into haem was then counted in an LKB 80,000 automatic gamma counter. None of the parameters measured showed a Normal distribution. Correlations were assessed using Spearman's rank correlation coefficient and difference between groups by the Mann–Whitmey U test (Siegel, 1956).

### RESULTS

### *Clinical stages and total marrow plasma-cell counts*

Total plasma-cell population was estimated in only 12 patients. Three in Stage I (Durie and Salmon, 1975) had between 0·10 and $3{\cdot}34 \times 10^{11}$; 2 in Stage II had 0·54 and $12{\cdot}58 \times 10^{11}$; and 7 in Stage III had between 1·81 and $5{\cdot}73 \times 10^{11}$. The percentage of plasma cells in the marrow ranged from 1% to 70% and did not appear to be related to the clinical staging.

### *Renal function*

Six patients had a blood-urea concentration of > 10 mM and 5 had a serum creatinine of > 200 $\mu$M. There was a significant negative correlation between haemoglobin concentration and serum creatinine ($r_S = -0{\cdot}52$, $P < 0{\cdot}05$) but no correlation between the concentrations of haemoglobin and blood urea ($r_S = -0{\cdot}31$, $P < 0{\cdot}1$). Neither haemoglobin concentration, marrow iron turnover,

TABLE I.—*Haematological and biochemical variables in 24 patients with myelomatosis*

| | Normal reference values | Myelomatosis patients Mean | Range |
|---|---|---|---|
| Haemoglobin concentration (g/dl) | 11·9–17·1 | 10·7 | 7·1–15·3 |
| Mean corpuscular volume (fl) | 86–104 | 93 | 81–116 |
| Mean corpuscular haemoglobin (pg) | 27·0–32·0 | 30·6 | 26·5–37·6 |
| Blood urea (mM) | 2·5–7·5 | 11·3 | 3·4–58·5 |
| Serum creatinine (μM) | 60–120 | 202 | 70–1260 |
| Serum iron (μM) | 12–34 | 12·4 | 1·5–33·0 |
| Total iron binding capacity (μM) | 45–70 | 51·8 | 20·5–95·0 |
| Transferrin saturation (%) | 25–50 | 24·0 | 7·3–53·2 |
| Serum ferritin (μg/l) | 14–370 | 197 | 27–745 |
| Serum folate (μg/l) | 2·1–21 | 5·8 | 1·9–13·1 |
| Red cell folate (·g/l) | 120–600 | 313 | 60–752 |
| Serum paraprotein (g/l) | | | |
| IgG (10)* | — | 38·8 | 16·7–59·1 |
| IgA (7) | — | 31·0 | 7·7–57·0 |
| IgM (1) | — | 8·3 | – |
| Serum immunoglobulins (g/l) | | | |
| IgG (14) | 4·5–16·0 | — | 3·3–58·5 |
| IgA (18) | 1·2–4·0 | — | <0·6–9·0 |
| IgM (23) | 0·45–1·8 | — | <0·2–2·0 |

* No. of patients in brackets.

haem synthesis nor the *in vitro* response of the marrow to Epo were significantly different between patients with blood urea concentrations above and below 10 mM.

*Immunoglobulins*

The serum paraprotein levels of the patients are summarized in Table 1. Thirteen patients had immunoparesis and their immunoglobulins were below the normal laboratory reference value shown in Table I. The serum paraprotein level was significantly correlated with the percentage of plasma cells in the marrow ($r_S = 0{\cdot}70$, $P < 0{\cdot}01$) with the total marrow plasma cells ($r_S = 0{\cdot}76$, $P < 0{\cdot}01$), total blood volume ($r_S = 0{\cdot}63$, $P < 0{\cdot}05$) and with serum folate concentration ($r_S = -0{\cdot}73$, $P < 0{\cdot}001$). In the patients with light-chain myeloma, urinary concentration of light chains varied between 0·4 and 12·1 g/l (mean 5·0 g/l).

*Haematological status*

The haematological and biochemical statuses of the patients are summarized in Table I. Nine of the patients had a haemoglobin concentration of < 10 g/dl. There was a significant negative correlation between haemoglobin concentration and serum paraprotein ($r_S = -0{\cdot}56$, $P < 0{\cdot}05$), total marrow plasma cells ($r_S = -0{\cdot}62$, $P < 0{\cdot}05$) and total blood volume ($r_S = -0{\cdot}71$, $P < 0{\cdot}01$). Haemoglobin concentration was significantly correlated with the percentage of plasma cells in the marrow ($r_S = -0{\cdot}43$, $P < 0{\cdot}05$).

Stainable iron was present in the marrow of all the patients, but no sideroblasts or megaloblasts were seen. Four patients had both low serum iron and total iron binding capacity, but a low transferrin saturation was found only in 3 patients. No patient had a serum ferritin < 15 μg/l, the level conventionally denoting iron deficiency. One patient had a low serum folate (1·9 μg/l) and red-cell folate of 60 μg/l.

*Serum erythropoietin*

The serum Epo concentrations of the uraemic patients were all within the normal range of 3–30 mu/ml (Fig. 1). Four anaemic patients without renal failure had raised serum Epo concentrations appropriate to the degree of anaemia.

FIG. 1.—The relationship between serum erythropoietin and blood urea in anaemic (○) and non-anaemic (●) patients with myelomatosis. Shaded area represents normal range.

*Blood volume and ferrokinetic studies*

The comparison of blood volume and ferrokinetic measurements in normal subjects and patients is summarized in Table II. The mean blood volume was significantly higher in myeloma patients (68·6 ml/kg) than in normal subjects (63·0 ml/kg) though the degree of haemodilution implied by this relatively small difference would not be sufficient by itself to account for the difference in haemoglobin concentration between the groups. Total marrow iron turnover is significantly lower in myeloma patients (81 μmol/l/d) than in normal subjects (115 μmol/l/d) but there was no difference in the degree of ineffective iron turnover. The mean marrow iron turnover in Stage I patients was 101·5 μmol/l/d, in Stage II patients 82·0 μmol/l/d and in Stage III patients 58·0 μmol/l/d. These differences are not however, statistically significant, possibly because of the small numbers involved. The mean red-cell lifespan was similar in the two groups and there was no difference in non-erythroid iron turnover. The total erythroblast population was measured only in 5 of the normal subjects, and a range of $2{\cdot}06\text{–}5{\cdot}67 \times 10^9$ cells/kg

TABLE II.—*Blood volume and ferrokinetic measurements in* 20 *normal subjects and* 16 *patients with myelomatosis* (*mean and range*)

| | Normal | Myeloma | U | *P* |
|---|---|---|---|---|
| Plasma volume (ml/kg) | *39·6*<br>31·9–50·7 | *49·8*<br>38·3–62·9 | 42 | <0·002 |
| Red-cell volume (ml/kg) | *25·1*<br>20·4–33·0 | *18·8*<br>13·2–23·9 | 17 | <0·002 |
| Blood volume (ml/kg) | *63·0*<br>52·3–74·7 | *68·6*<br>535–82·3 | 83 | <0·05 |
| Marrow iron turnover (MIT) (μM/day) | *115*<br>73–145 | *81*<br>33–135 | 65 | <0·002 |
| Ineffective iron turnover (% of MIT) | *23*<br>13–34 | *21*<br>8–39 | 126 | n.s. |
| Mean red-cell lifespan (days) | *96*<br>67–153 | *98*<br>57–166 | 157 | n.s. |
| Non-erythroid iron turnover (μM/day) | *29*<br>5–57 | *23*<br>1–52 | 134 | n.s. |
| Total erythroblast count ($\times 10^6$/kg) | *4·1*<br>2·1–5·7 | *3·3*<br>1·0–9·4 | 20 | n.s. |
| Erythroblast iron uptake (μM/$10^{12}$ cells/day) | *2198*<br>1503–3167 | *1939*<br>884–4452 | 18 | n.s. |

FIG. 2.—$^{59}$Fe incorporation into haem in patients' marrow cultures after 4 days incubation in basal medium or with the addition of 0·5 $\mu$/ml erythropoietin. Shaded area represents normal range.

was obtained. In 4/12 myeloma patients where it was possible to make this measurement, values below $2\times10^9$ cells/kg were found and 3 of these were patients with Stage III disease. In the myeloma patients haemoglobin concentration was significantly correlated with total marrow erythroblast population ($r_S=0{\cdot}60$, $P<0{\cdot}05$).

In normal subjects (Cavill & Ricketts, 1980) there is a significant negative correlation between marrow iron turnover and red-cell lifespan, and this is true for the present normal group ($r_S=-0{\cdot}73$, $P<0{\cdot}001$) and for patients with myelomatosis ($r_S=-0{\cdot}88$, $P<0{\cdot}001$) but, as can be seen from Fig. 2, the marrow iron turnover in myeloma patients is lower than normal, for the corresponding red-cell lifespan. There is no difference in erythroblast iron uptake *in vivo* between normal and myeloma subjects.

### In vitro *erythroblast iron metabolism*

The erythroblast iron uptake in normal subjects ranged from 7 to 30 $\mu$mol/$10^{12}$ cells/h (mean 15 $\mu$mol) and this was increased in 8 of the 17 patients studied. The cytosol iron incorporated into haem in the normal subjects was 55–84%. In 3 patients with high erythroblast iron uptake the percentage incorporation into haem was somewhat less, but the absolute amount incorporated was normal. There was no significant abnormality of iron incorporation into ferritin or soluble iron complexes.

### In vitro *marrow responses to erythropoietin*

The effect of erythropoietin on haem synthesis after 4 day culture of marrow samples is shown in Fig. 3. In the absence of added Epo there was no significant difference between the patients (3·9 $\mu$mol/$10^{12}$ cells) and normal controls (4·3 $\mu$mol/

FIG. 3.—The relationship between marrow iron turnover and mean red-cell lifespan in normal subjects ○ and patients with myelomatosis ●.

$10^{12}$ cells ) (U = 82, $P < 0 \cdot 1$), though there was greater variation between patients than between controls. With the addition of 0·25 u/ml Epo, the mean $^{59}Fe$ incroporation in haem was 10·4 $\mu$mol/$10^{12}$ cells in the patients compared to 13·1 $\mu$mol/$10^{12}$ cells in the control group (U = 53, $P < 0 \cdot 02$) and again haem synthesis was more variable in the patients than in the controls, being above abnormal in 2 and subnormal in 9. The mean percentage increase in the incorporation of $^{59}Fe$ in haem on stimulation with Epo was 166% in the patients and 226% in normal subjects (U = 69, $P < 0 \cdot 1$). There did not appear to be any relation between the marrow response to Epo and haemoglobin concentration, blood urea, serum paraprotein concentration, total plasma cell numbers, marrow iron turnover or total marrow erythroblast numbers.

### DISCUSSION

Anaemia is a common feature of myelomatosis, being found in 90% of patients seen in Edinburgh before 1959 (Innes & Newall, 1961) and 62% of a series seen in the Mayo Clinic up to 1971 (Kyle, 1975). 237 out of 485 patients entering the third MRC myelomatosis trial had an initial haemoglobin concentration of 10 g/dl or less (MRC Working Party, 1980*b*). The anaemia has usually been described as normochromic or normocytic (Innes & Newall, 1961; Kyle, 1975) and may be the result of haemodilution due to an expanded plasma volume (Bjorneboe & Jensen, 1969; Alexanian, 1977) though this is by no means universally agreed (Cline & Berlin, 1962; Hansen & Drivsholm, 1978). It has been suggested that the haematological changes in myelomatosis result from displacement of normal marrow by an abnormal growth of plasma cells (Innes & Newall, 1961; Kyle, 1975). In addition, megaloblastic erythropoiesis due to both folate and B12 deficiency has been found in a high proportion of cases (Hoffbrand *et al.*, 1967) and about a quarter of patients have been estimated to be iron deficient (Hansen, 1978; Birgens *et al.*, 1979). Sideroblastic changes have been described in a few patients with myelomatosis (McGibbon & Mollin, 1965; Dacie & Mollin, 1966) and this feature may precede the occurrence of myelomatosis in some cases (Catovsky *et al.*, 1971). In some patients on long-term chemotherapy for myelomatosis, sideroblastic anaemia may precede the onset of acute leukaemia (Khaleeli *et al.*, 1973). Cline & Berlin (1962) postulated an erythroid hypoplasia in myelomatosis which was not related to the degree of marrow infiltration. However, their evidence, based largely on the plasma iron turnover, was obtained from the investigation of treated patients. Hansen *et al.* (1977) found depressed erythropoietic activity in anaemic patients with myelomatosis and assumed that this was due to renal failure, as venous haematocrit was correlated with glomerular filtration rate. About a quarter of the patients studied by Cline & Berlin (1962), Hansen (1978) and Birgens *et al.* (1979) had no stainable marrow iron, but Hansen felt that the normal transferrin saturation and sideroblast count in his patients probably indicated an adequate supply of iron to the erythroid marrow.

Only 3 of our patients had a low transferrin saturation, but all of them had stainable iron in their marrow and serum ferritin concentrations within or above the normal range, indicating that iron deficiency is not an important factor in the pathogenesis of their anaemia. We were not able to confirm the findings by Hoffbrand *et al.*, (1967) that folate deficiency and megaloblastic erythropoiesis were common in patients with myelomatosis, but more than half of their cases had already received chemotherapy or radiotherapy, and many of them had light-chain myeloma. In our patients the negative correlation between serum folate and paraprotein concentration suggests a relationship with the disease process, though only one patient had a pathologically low serum folate level.

Anaemia in myeloma patients with normal renal function suggests that extra-renal factors may be an important factor in its aetiology. None of our patients had subnormal serum levels of Epo, and in patients with normal renal function, increased concentrations appropriate to the reduced haemoglobin level were found (Fig. 1). These findings differ from those of Hansen *et al.* (1977), who found that most of their patients had no detectable circulating Epo. This may have been due to the low sensitivity of their Epo assay, based on post-hypoxic protein-starved mice. The mean blood volume of our myeloma patients was 68·6 ml/kg, compared to 63·0 ml/kg in normal subjects, and although this difference is statistically significant, its physiological significance may not be great. However, the negative correlation between haemoglobin concentration and blood volume in the patient indicates that haemodilution is probably a factor in the pathogenesis of their anaemia. The correlation between paraprotein concentration and blood volume suggests that plasma volume expansion is directly related to the protein abnormality. There is no relationship between blood volume and paraprotein type in our patients. The present findings are in agreement with those of Alexanian (1977) and Bjorneboe & Jensen (1969) However, Cline & Berlin (1962) and Hansen & Drivsholm (1978) found normal blood volumes in their patients. There were not only differences in methodology which might account for the disagreement, but most of the patients studied by Cline and Berlin had already been given extensive treatment and are thus not comparable to the present untreated group. The negative correlation between serum paraprotein concentration and haemoglobin concentration might be due partly to the effect of haemodilution, or might simply reflect an association between anaemia and the primary disease process.

Durie & Salmon (1975) proposed a clinical staging scheme based on correlations between tumour mass and certain presenting clinical features. The total tumour cell cell number was derived from measurements of immunoglobulin synthesis rates by myeloma cells *in vitro* and synthetic rate of myeloma protein measured *in vivo*. There is some suggestion from our data that marrow iron turnover and erythroblast numbers may be related to clinical staging. The total plasma-cell population estimated by our method is somewhat lower than the figures expected from the studies of Durie & Salmon (1975) at each stage of the disease, though there is an increase with advancing stages and there is an overlap with their figures for Stage II patients. The discrepancy is probably accounted for by the necessary assumption in our calculations that the erythroblast: plasma-cell ratio is constant throughout the diseased tissues. Plasmacytomas would not be accounted for by our method and the calculations depend very much on the plasma-cell content of the sample aspirated, which may not, of course, represent the marrow as a whole.

Our myeloma patients showed a decreased rate of marrow iron turnover, while red-cell lifespan and ineffective erythropoiesis remained normal. This points to a significant impairment of the erythroid proliferative response to anaemia. The erythroblasts produced appear to behave normally. Although erythroblast iron uptake was increased in a few cases, haem synthesis was normal and no abnormal sideroblasts were seen. The factors controlling erythroid iron uptake are not understood. The only previous observations of increased uptake have been in patients with iron deficiency or secondary sideroblastic change (unpublished observations) neither of which are present in the cases studied here.

The Epo response of the late erythroid precursors in marrow cultures was similar in normal and myeloma groups as a whole, but the patients showed far more variability than the control subjects, and in many cases the total iron incorporated into haem after Epo stimulation was subnormal. It is difficult to judge from the present data whether decreased response to Epo may sometimes be important The reduction in total erythropoiesis measured by ferrokinetics, together with normal erythoblast iron uptake, suggests a reduced input from a precursor compartment, presumably due to a failure of maturation, though whether this is manifested at BFU-E or CFU-E level is not obvious. Except in patients with renal failure, the level of circulating Epo does not appear to be a limiting factor. In individual cases a superimposed folate deficiency may be important, and there appears to be a haemodilution factor related to the concentration of circulating paraprotein. The impression of a contracted erythroid marrow compartment in myeloma patients is similar to that found in patients with Hodgkin's disease (Al Ismail *et al.*, 1979) but in neither case is there any direct indication of the precise mechanism.

Dr W. C. Ting is a Leukaemia Research Fund Fellow.

### REFERENCES

ALEXANIAN, R. (1977) Blood volume in monoclonal gammopathy. *Blood*, **49**, 301.

AL-ISMAIL, S., CAVILL, I., EVANS, I. H., JACOBS, A., RICKETTS, C., TREVETT, D. & WHITTAKER, J. A. (1979) Erythropoiesis and iron metabolism in Hodgkin's disease. *Br. J. Cancer*, **40**, 365.

BIRGENS, H. S., HANSEN, O. P., HENRIKSEN, J. H. & WANTZIN, P. (1979) Quantitation of erythropoiesis in myelomatosis. *Scand. J. Haematol.*, **22**, 357.

BJORNEBOE, M. & JENSEN, K. B. (1969) Plasma volume, colloid osmotic pressure and gamma globulin in multiple myeloma. *Acta Med. Scand.*, **186**, 975.

CATOVSKY, D., SHAW, M. T., HOFFBRAND, A. V. & DACIE, J. A. (1971) Sideroblastic anaemia and its association with leukaemia and myelomatosis: A report of five cases. *Br. J. Haematol.*, **20**, 385.

CAVILL, I. & RICKETTS, C. (1980) Human iron kinetics. In *Iron in Biochemistry and Medicine, II.* (Ed. Jacobs & Worwood) New York: Academic Press.

CAVILL, I., RICKETTS, C. & JACOBS, A. (1977) Radioiron and erythropoiesis: Methods interpretation and clinical application. *Clin. Haematol.*, **6**, 583.

CLINE, M. J. & BERLIN, N. I. (1962) Studies of the anaemia of multiple myeloma. *Am. J. Med.*, **33**, 510.

DACIE, J. V. & MOLLIN, D. L. (1966) Siderocytes, sideroblasts and sideroblastic anaemia. *Acta. Med. Scand.*, **445**, (Suppl.), 237.

DURIE, B. G. & SALMON, S. E. (1975) A clinical staging system for multiple myeloma. *Cancer*, **36**, 842.

FAHEY, J. L. & MCKELVEY, E. M. (1965) Quantitative determination of serum immunoglobulins in antibody agar plates. *J. Immunol.*, **94**, 34.

HANSEN, O. P. (1978) Bone marrow studies in myelomatosis. *Scand. J. Haematol.* **21**, 265.

HANSEN, O. P. & DRIVSHOLM, A. (1978) Interrelationships between blood volumes, venous haematocrit and renal failure in myelomatosis. *Scand. J. Haematol.*, **20**, 461.

HANSEN, O. P., THORLING, E. B. & DRIVSHOLM, A. (1977) Serum erythropoietin in myelomatosis. *Scand. J. Haematol.*, **19**, 106.

HOFFBRAND, A. V., HOBBS, J. R., KREMENCHUZKY, S. & MOLLIN, D. L. (1967) Incidence and pathogenesis of megaloblastic erythropoiesis in multiple myeloma. *J. Clin. Pathol.*, **20**, 699.

INNES, J. & NEWALL, J. (1961) Myelomatosis. *Lancet*, i, 239.

JONES, B. M. & WORWOOD, M. (1975) An automated immunoradiometric assay for ferritin. *J. Clin. Pathol.*, **28**, 540.

KHALEELI, M., KEANE, W. M. & LEE, G. R. (1973) Sideroblastic anaemia in multiple myeloma. *Blood*, **41**, 17.

KRANTZ, S. B., GALLIAN-LANTIQUE, O. & GOLDWASSER, E. (1963) The effect of erythropoietin upon haem synthesis by marrow cells *in vitro*. *J. Biol. Chem.*, **238**, 4085.

KRANTZ, S. B., MOORE, W. H. & SAINTZ, S. D. (1973) Studies on red cell aplasia. V. Presence of erythroblast cytotoxicity in a G-globulin fraction of plasma. *J. Clin. Invest.*, **52**, 324.

KYLE, R. A. (1975) Multiple myeloma: Review of 869 cases. *Mayo. Clin. Proc.*, **50**, 29.

MCGIBBON, B. H. & MOLLIN, D. L. (1965) Sideroblastic anaemia in man: Observation on seventy cases. *Br. J. Haematol.*, **11**, 59.

MEDICAL RESEARCH COUNCIL (1980*a*) Report on the second myelomatosis trial after 5 completed years of follow-up. *Br. J. Cancer*, **42**, 813.

MEDICAL RESEARCH COUNCIL (1980*b*) Prognostic features in the third MRC myelomatosis trial. *Br. J. Cancer*, **42**, 831.

NAPIER, J. A. F. & EVANS, J. (1980) Erythropoietin assay using foetal mouse liver cell cultures: A modified technique using semi-automatic harvesting of I-deoxyuridine labelled erythroblasts. *Clin. Lab. Haematol.*, **2**, 13.

RICKETTS, C., JACOBS, A. & CAVILL, I. (1975) Ferrokinetics and erythropoiesis in man. The measurement of effective erythropoiesis, ineffective erythropoiesis and red cell lifespan using $^{59}$Fe. *Br. J. Haematol.*, **31**, 65.

SIEGEL, S. (1956) *Nonparametric Statistics for the Behavioural Sciences.* Tokyo: McGraw-Hill.

TEALE, F. W. J. (1959) Cleavage of the haem protein link by acid methylethylketone. *Biochim. Biophys. Acta*, **35**, 543.

TENNANT, G. B. (1977) Continuous flow automation of *Lactobacillus casei* serum folate assay. *J. Clin. Pathol.*, **30**, 1168.

YOUNG, D. S. & HICKS, J. M. (1965) Method for the automatic determination of serum iron. *J. Clin. Pathol.*, **18**, 98.